# Development of Hydroxyapatite as a Bone Implant Biomaterial for Triggering Osteogenesis

**DOI:** 10.1055/s-0045-1809312

**Published:** 2025-05-27

**Authors:** Yusuf Alif Pratama, Honey Dzikri Marhaeny, Fani Deapsari, Aniek Setiya Budiatin, Mahardian Rahmadi, Andang Miatmoko, Muhammad Taher, Junaidi Khotib

**Affiliations:** 1 Doctoral Program of Pharmaceutical Science, Faculty of Pharmacy, Universitas Airlangga, Surabaya, Indonesia; 2 Department of Pharmacy Practice, Faculty of Pharmacy, Universitas Airlangga, Surabaya, Indonesia; 3 Department of Pharmaceutical Sciences, Faculty of Pharmacy, Universitas Airlangga, Surabaya, Indonesia; 4 Department of Pharmaceutical Technology, Kulliyyah of Pharmacy, International Islamic University Malaysia, Pahang, Malaysia

**Keywords:** natural hydroxyapatite, immune response, bone, neglected disease, bone remodeling

## Abstract

Over the past decade, the occurrence of bone defects has seen a notable rise. In both developed and developing nations, their prevalence tends to increase in parallel with population density and levels of physical activity. Various therapeutic approaches have been implemented to address bone fractures, focusing on preventing infections, promoting faster healing, and restoring normal bone function. Among these, bone grafting—a surgical technique involving the use of biomaterials—remains a widely utilized method for bone replacement. This review aims to identify biomaterials that have biocompatibility with bone, osteoinductive, and osteoconductive properties so that they can trigger good osteogenesis. This review is based on a compilation of publications from various databases related to factors affecting the process of bone ossification. This study also evaluates the characteristics of hydroxyapatite biomaterials that play a role in inducing osteogenesis. The phosphate/calcium ratio close to 1.67, porosity in the range of 40 to 60%, pore diameter of 200 to 900 nm, and crystallinity of 40 to 60% will help the osteogenesis to perform well. The results of this study highlight the advantages of hydroxyapatite in terms of its osteoconductive, osteoinductive, and osteointegrative properties, which can trigger osteogenesis.

## Introduction

When a bone is fractured, the first step is the formation of a hematoma in the fractured part of the bone. Several inflammatory mediators, including cytokines such as interleukin-1 (IL-1), IL-6, IL-18, and tumor necrosis factor-α (TNF-α), increase significantly in the first few days after bone fracture. 1 Proinflammatory mediators have a chemotactic effect on other inflammatory cells; thus, after vascular trauma in the fracture area, hypoxia occurs, and osteocytes in the fracture area experience necrosis. Macrophages phagocytose the necrotic region and facilitate the regeneration stage by releasing important signaling factors, healing factors such as bone morphogenetic proteins (e.g., BMP-2, −5, −7), basic fibroblast growth factor (bFGF), transforming growth factor-β (TGF-β), platelet-derived growth factor (PDGF), and insulin-like growth factor. These growth factors are responsible for the migration, recruitment, and proliferation of mesenchymal stem cells (MSCs) and the differentiation of angioblasts, chondroblasts, fibroblasts, and osteoblasts. 2 3

Various therapeutic strategies have been applied to overcome bone fractures with the aim of preventing bone infections, promoting faster fracture healing, and restoring physiological functions. Bone replacement procedures, commonly referred to as bone grafting, involve surgical procedures that use specific materials. 4 There are several types of bone grafting methods, such as autografting, allografting, xenografting, and alloplasty. Autograft, which is the gold standard in bone grafts, 5 is a technique in which a bone graft from the patient's healthy bone is transplanted into the patient's bone defect. Allografting is the process of retrieving and transferring bone from species different from humans, such as bovine, equine, and porcine. In addition to using materials from living beings, bone grafting techniques can use synthetic biomaterials. Biological materials are also known as alloplastics. These biomaterials can replace and repair tissues, organs, and even functions of the body, such as hip joint prostheses, sutures, screws, bone plates, artificial blood vessels, muscle stimulators, and artificial stapes. 6 Biomaterials that can be used as bone graft materials include hydroxyapatite (HA), bioactive glass, and aluminum oxide. 7 8

Implants are often used as medicinal carriers for bone. Biomaterials that are used today include medical bioceramic materials, medical polymers, medical composites, and nano-artificial bones. Ideal bone implants have biological activities that induce bone tissue regeneration, prevent bacterial infections, do not evoke a sustained inflammatory response, and have good mechanical strength. 9 10 Based on the type, implants are divided into biodegradable and nonbiodegradable implants. The use of biodegradable implants, such as ceramic and polymeric implants, has several advantages, such as higher porosity, greater biocompatibility, controlled biodegradation, and the production of nontoxic degradation products while maintaining good mechanical properties. Some studies have reported osteoconductive properties of biodegradable implants. 11 12 In comparison, the nonbiodegradable materials, commonly made of stainless steel, titanium, and cobalt-chromium alloy, have a major disadvantage, such as the second surgery to take the implant out. Thus, the development of biodegradable materials to conduct osteogenesis is a must.

HA (Ca _10_ (PO _4_ ) _6_ (OH) _2_ ), a calcium phosphate bioceramic, is a well-known material used in bone reconstruction. HA is mostly used in medicine to fill and construct bones and teeth. Generally, HA is mixed and coated with minerals and lacquer materials to improve its brittleness and strength. 13 HA can assist the bone-healing process owing to its good osteoconductive and osteoinductive properties. In addition, HA has several advantages, including good biological compatibility, affinity, bioactivity, and osteointegration, mainly due to its biodegradable characteristics. There are no known incidences of toxicities, both locally and systemically. 14 15

Tricalcium phosphate (TCP) and biphasic calcium phosphate (BCP) are the other common types of calcium/phosphate ratio. HA gives the optimal biocompatibility, stability, osteoconductivity, and osteoinductivity. It makes HA superior to TCP. Meanwhile, the BCP is a mixture of HA and TCP. Other biomaterials, such as collagen and chitosan as natural polymers, are good materials to exchange the extracellular matrix for cartilage, while polylactic acid (PLA), polyglycolic acid, and poly(lactic-co-glycolic) acid are good materials for bone fixation. Those two materials are not calcium/phosphate sources, which are not the main source for bone osteogenesis.

This review aimed to deepen the identification of HA, a biodegradable material that has good biocompatibility with bone and is osteoinductive and osteoconductive while encouraging osteogenesis.

## Types, Characteristics, and Preparation Method of Natural Hydroxyapatite

Based on its type, HA can be of natural or synthetic origin. One example of naturally derived HA is bovine HA (BHA), which is derived from bovine bone. Bovine bone is preferred because it is similar to human bone HA and is a scaffold with more osteoconductive properties than synthetic HA. 16 17 The BHA has a pore diameter of between 250 and 450 μm, and the porosity varies from 30 to 70%. Its porosity is greater than that of synthetic HA, which can be nanosized. High porosity causes BHA to coalesce easily with bone tissue through physical and chemical bonding. In addition, BHA is more stable against radiation exposure than other biomaterials. 18 Natural HA has excellent biocompatibility, high bioactivity for bone tissue reconstruction, low biodegradation, and osteoconductive properties. Good porosity and insoluble and nonabsorbable properties make natural HA an ideal material for new bone formation. 19 20

Natural HA can be isolated from a variety of sources, such as bovine bones, human bones, camel bones, horse bones, pig bones, fish scales, fish bones, cuttlefish bones, salmon, trout, cod bones, shells, plants, algae, and minerals. In general, the manufacture of natural HA from bovine bone begins when the bovine femur is placed in boiling water for 1 hour to defeat and facilitate the removal of macroscopic contamination. The samples are then immersed in acetone for 2 hours, rinsed, and dried. Dried bones can be crushed into small particles, and size distribution within a certain size range can be determined. The calcination process is performed in an oven at high temperatures to remove the organic components. 21 Another recent nonhazardous method was introduced owing to longer conventional processes and environmentally unfriendly waste disposal. This method reduces the use of harmful solvents, such as hexane, because of their carcinogenic effects; hexane is often used to remove fat, and hydrogen peroxide is used as a protein-breaking agent. 16 The method used in this sequence removes the sponge and marrow from the bone marrow. The bones are then boiled in a high-pressure tank, dried, and immersed in absolute ethanol to remove fat and protein residues. Calcination is then performed at high temperatures, and the product is powdered. 16

## Comparison of Characteristics and Effectiveness of Natural and Synthesized Hydroxyapatite

Natural HA is used as a bone filler because the inorganic phase in animal bones has characteristics similar to those of human bone. The main constituents of natural HA are Ca and P, with minor amounts of Na, Mg, O, and C. These components can accelerate resorption and reversal during the remodeling process, followed by synthetic HA. 22 The higher concentration of Mg in natural HA than in synthetic HA improves several factors in the process. Mg implants have long been used to induce bone-cell formation. 23 However, synthetic HA has the disadvantage that it does not contain microcomponents of Mg, Na, and CO _3_^2−^ groups. 24

The preparation of HA from natural HA showed that proper characteristics for the proliferation and differentiation of osteoblasts resulted in similar attributes to bone composition in humans. 25 The high Ca/P ratio in natural HA (> 1.67) reflects the composition of human bones. However, a high Ca/P ratio is undeniably due to the presence of compounds other than HA. 26 The Ca/P ratio of synthetic HA offers precise control, as well as uniform pore diameter, crystal size, shape, and porosity, while natural HA will give various characteristics. 27 28

The concentration of bicarbonate ions in natural HA determines the bioactivity of HA. The bioactivity of bicarbonate ions is indicated by increased protein adsorption in conjunction with increased adhesion, proliferation, and osteogenic differentiation of MSCs. 29 In addition, carbonated HA is known to increase osteoblast proliferation and alkaline phosphatase (ALP) concentration *in vitro* . However, in some HA synthesis methods, it is common to increase the carbonate content. Commonly, CO _2_ in the atmosphere is dissolved in a suspension to obtain carbonate ions. 30

Based on Khotib et al, 27 the use of BHA as natural HA can increase the number of osteoclast and osteoblast on the 14th, 28th, and 42th day. Osteoclast arose from 0.66 ± 0.57 to 2.50 ± 0.70 in the control group, while in the BHA group, osteoclast arose from 2.66 ± 1.54 to 3.00 ± 0.70. For the number of osteoblasts, in the control group, osteoblast arose from 102.00 ± 22.27 to 188.00 ± 91.92, while in the BHA group, osteoblast arose from 202.33 ± 27.42 to 241.50 ± 42.42. It was a significant increase with *p* < 0.05.

In addition to coming from living organisms, HA made at the nanoscale helps the bone regeneration process. Nowadays, the development of nano-HA has become very massive. 31 32 One of the purposes of HA nanosynthesis is to facilitate mass production. The physicochemical properties of HA depend on the preparation method used. 33 Overall, the synthesis of HA showed low crystallinity, high porosity, and a large surface area. 34 In addition, a study reported that the particle surface morphology, size distribution, and surface hydration level of HA nanoparticles have a significant effect on osteogenic properties. Nano-HA can accelerate bone filling and provide high-quality bone growth results. Meanwhile, the combination of platelet-rich fibrin can increase the level of bone formation compared with nano-HA and controls. 35

## Factors Affecting Osteogenesis

Bone regeneration is a complex, gradual, and dynamic process. This process is initiated by stem cell migration and induction, which subsequently proliferate, differentiate, and induce matrix deposition. Immunomodulation and vascularization play crucial roles in efficient bone repair. 36 Thus, the materials used in the process of healing bone defects have at least one effect on increasing immune regulation and the effectiveness of angiogenesis and osteogenesis. Osteogenesis is the dominant process during bone formation and is characterized by early bone development and terminalization of the extracellular matrix. 37 Osteoblasts play an important role in the mineralization of osteoid, which is the extracellular matrix of the bone tissue. Osteoblasts are derived from MSCs, and their differentiation from these stem cells is called osteoblastogenesis. 38 As MSCs differentiate into osteochondrocyte precursors, several molecules (including Runx2, β-catenin, or Dlx3/5/6) are needed that can induce such precursors into immature osteoblasts. Osteoblast maturation is mediated by several proteins, including osterix, NFAT (nuclear factor of activated T cells), and β-catenin. These factors activate osteoclasts, osteopontin, and osteonectin during the final differentiation of mature osteoblasts into osteocytes. 38

The Ca/P ratio in bone plays an important role in osteogenesis. The molar and mass ratios of Ca and P in bone are at 1.7 and 2.2, respectively. The mass ratio of Ca/P in the bone varies between 1.9 and 2.4. Mineralization is required during bone development, which strengthens the collagen-bound tissue, forming the organic matrix. Without the mineralization phase, there will not be sufficient use of Ca and P ions in the extracellular region until the deposition becomes an organic matrix. 39

Calcium plays an important role in bone formation. The exposure of osteoblasts to Ca ions can stimulate proliferation and differentiation. However, a large increase in calcium concentration can also inhibit osteoclastogenesis. *In vivo* studies have shown that increased extracellular calcium levels can stimulate bone formation and inhibit bone resorption. 39 Cellular mechanisms of calcium ions can control osteoblast function and bone formation stimulation mediated by Ca-sensitive receptors (CaSRs). CaSRs also play important roles in chondrogenic plate growth and bone development. 40

The role of phosphorus in the process of bone formation and mineralization lies in its ability to increase the production of bone matrix by osteogenic cells. Several studies have reported that hypophosphatemia affects the ability of the body to produce active factors involved in bone formation and systemic phosphorus homeostasis. 39 Osteoclasts exposed to phosphorus can lead to changes that decrease bone resorption activity. Such changes include the differentiation of preosteoclasts and stimulation of apoptosis in mature osteoclasts. 40

The surface characteristics of a scaffold or implant play an important role in controlling cell reactions and improving tissue efficiency. The porous structure is used for cell adhesion and new bone formation. 41 However, it can also play a role in nutrient transport and disposal of cell metabolites. Another role played by the presence of pores in a scaffold or implant material is that spherical pores can hold larger amounts of tissue and have a higher compressive strength. It is, therefore, evident that the considerable pore structure provides advantages as a place of attachment of bone cells to which nutrients and oxygen are distributed. However, too large a pore size (> 1000 μm) is not desirable in the process of bone growth. 42 Studies that compare the effect of large pore size on bone growth ability have found that larger pore sizes (200–400 μm) provide a place for tissue to invade compared with smaller pore sizes (50–100 μm). Another study recommended a minimum pore size of 100 μm for effective function in new bone formation. 43

The presence of a macroporous structure also affects the distribution of fluid and fluid pressure in the scaffold. Fluid dynamics in the porous scaffold has a direct impact on adhesion, migration, proliferation, and cell differentiation, as well as on tissue growth. The high penetration strengthens the ability of the material to induce new bone formation. 44 With an increase in the pore size, the liquid flow rate increases owing to an increase in the diameter of the interconnection gap. 42 Another influential factor is that the more surfaces that are used as a place for new bone formation, the better. This was shown by Yoshikawa et al 45 ; a scaffold with a hole in the middle provides an opportunity for new tissue to be formed not only on the periphery but also on the inside.

When the damaged tissue is rebuilt, sufficient nutrients and oxygen are required for the formation of new cells to initiate cell proliferation, maturation, and differentiation. It is known that a cell can survive with a blood vessel that provides nutrients and oxygen and transports metabolic products and effluents from the cell. The presence of circulating blood vessels can help stabilize the body temperature and prevent pH imbalances. Vascular formation occurs owing to angiogenic stimuli. This process occurs in several stages, including vasculogenesis, angiogenesis, and arteriogenesis. 46 47 The three processes take place sequentially, one of them after the presence of an incoming Ca ^2+^ signal. Incoming Ca ^2+^ can be affected by the stimulation of growth factors, such as vascular endothelial growth factor (VEGF), bFGF, PDGF, epidermal growth factor, stromal-derived factor-1α (SDF-1α), and angiopoietin-regulating endothelial cells. This causes the blood vessels to contribute significantly to homeostasis in an organism. Therefore, induction of angiogenesis is fundamental to efficient new tissue formation. Similar to other cells, bone cell formation requires the induction of new blood vessel formation. 46 48

Growth hormone is the main regulator that controls postnatal bone growth owing to its ability to stimulate the differentiation and proliferation of chondrocytes at the site of long bone growth. 49 In a study conducted by Laron and Klinger, 50 bone growth biomarkers decreased after the deletion of the growth hormone receptor. In addition, a clinical study by Ohlsson et al 49 reported decreased bone mineral density and concentration in patients with growth hormone deficiency. A study by Cool et al 51 shows that growth hormone influences hematopoietic and mesenchymal progenitor cells. Knockout of the growth hormone receptor causes a decrease in calcium accumulation and expression of Cbfa-1, type I collagen, osteopontin, and osteocalcin (OCN), thereby affecting osteogenesis.

## Mechanism of Natural Hydroxyapatite in Osteogenesis

Osteogenesis is the process of differentiation of human MSCs into osteoblasts due to certain stimulations characterized by an increase in ALP activity and formation of nodules containing type I collagen, OCN, osteonectin, bone sialoprotein, and osteopontin. 52 Cell proliferation and differentiation are common processes involved in bone tissue formation and regeneration. During bone formation, osteoblasts form type I collagen and proteoglycans in the bone matrix (osteoid) and secrete large amounts of ALP. 53 ALP is an enzyme located on the outer membrane of osteoblasts that functions as the main regulator of bone mineralization. Therefore, ALP is an important biomarker of bone formation. 54 ALP breaks phosphate bonds into free phosphate ions that react with calcium ions to form calcium phosphate bonds to form HA. This HA formed later accelerates calcification and bone mineralization. 53

HA can induce endothelial cell proliferation and maintain morphology and biochemistry associated with endothelial cell function. The utilization of HA, with its osteoconductive properties, can induce new vascular growth and mesenchymal apex cells to proliferate and differentiate into osteoblasts. HA also facilitates osteoblast migration to the surface, accelerating the bone healing process. 55 The pore structure of HA is an important aspect related to pore interconnectivity, as it can increase vascularization and, therefore, increase the supply of oxygen and nutrients. 56 The presence of calcium and phosphorus ions in HA also induces osteoblast differentiation. 57 HA is a biomaterial with a porous structure that can be used to induce osteogenesis and angiogenesis by promoting tissue growth in its pores. 44 Li et al 42 showed that HA with a pore size of 750 to 900 μm has a greater ability to induce osteogenesis and angiogenesis, as evidenced by an increase in the percentage of new bone formation area and density of new blood vessels.

The ability of HA to induce osteogenesis and angiogenesis is also evidenced by an increased expression of FGF2 and VEGF compared with the control group, number of osteoblasts and osteoclasts, expression of receptor activator of nuclear factor kappa-β ligand (RANKL), osteoprotegerin, OCN, and ALP. 53 Some studies mention that the activity of natural HA is greater than that of synthetic HA. FGF-2 is a potent mitogenic factor found in various cell types, including fibroblasts and osteoblasts. FGF-2 can induce angiogenesis through autocrine and paracrine factors that stimulate endothelial cell proliferation and migration, along with increased expression of potassium, growth factors, and integrins involved in angiogenesis. These molecules are important for the proliferation phase of bone repair. 58 In addition, HA is able to induce increased VEGF expression compared with the control group. VEGF is a powerful angiogenic factor stimulated by FGF-2. 59 These factors can stimulate or inactivate the migration, proliferation, and differentiation of endothelial cells during the formation of new blood vessels. VEGF also initiates angiogenesis through neovascularization, which stimulates the migration and recruitment of MSCs to defective bone areas and induces osteoblast differentiation along with BMP-2. 59 In addition, there was increased ALP expression compared with that in the control group. This demonstrates that an increase in ALP indicates bone calcification when the mineralization phase of bone formation progresses faster and bone healing improves. 53

A study by Anghelescu et al 60 showed that BHA could exert a significant influence on angiogenesis by increasing the expression of endothelial cell adhesion molecules (PECAM-1/CD31) and VEGF. Cluster of differentiation 31 (CD31) or PECAM-1 plays an important role in removing neutrophils from the body. These molecules are found on the surface of platelets, monocytes, neutrophils, and some types of T cells. 61 In this study, it was found that the presence of a bone graft with BHA material increased the number of blood vessels measured through CD31 and higher VEGF markers. This is due to the osteoconductive properties of BHA. 60

In addition to being a biomaterial that functions well in osteogenesis, HA can also be used as a carrier and combined with other materials to improve its osteoinductive, osteoconductive, and osteointegrative functions. Li et al 62 stated that the ability to release SDF-1 is a good bioactivity marker. SDF-1 is a chemokine that repairs damaged tissue. In addition, SDF-1 mediates cell migration to the cellular level and recruits migrating and settled stem cells *in vivo* . 63 SDF-1 placed in the HAp/PLA membrane releases SDF-1 to induce endogenous cell recruitment, immunomodulation, angiogenesis, and osteogenesis. 62 Other materials that have been used in several studies are ellagic acid, MSCs, coralline blocks, carbon nanofibers, silver nanoparticles, silver oxide, collagen, chitosan, sodium alginate, and glutaraldehyde. 17

## Mechanism of Natural Hydroxyapatite in Immune Response-Induced Osteogenesis

The immune system is important for new bone formation. Understanding the interaction between immunity and bone regeneration is important, especially in the context of bone implants and the healing process after fractures or surgery. Several studies have demonstrated the importance of the immune system in regulating osteogenesis, particularly during the healing process following injury or surgical intervention. The immune response is activated immediately following injury or bone implant placement and can be divided into several phases, beginning with acute inflammation. 64 The early inflammatory phase is critical to the process of osteogenesis. Proinflammatory cytokines such as TNF-α and IL-1 are released, attracting neutrophils and more macrophages. Although inflammation is considered negative, it serves an important dual role in osteogenesis: clearing debris and pathogens and facilitating the transition to the reparative phase. Immune cells promote the expression of osteogenic factors that stimulate osteoblast activity, which is critical for new bone formation. As the inflammatory response declines, the body transitions to the reparative phase characterized by anti-inflammatory cytokines such as IL-10 and TGF-β. 65 Cytokines can help MSCs become osteoblast. However, infection can lead to prolonged inflammation and harmful effects on bone regeneration. This response can cause degenerative diseases such as osteoporosis and osteoarthritis. 66

Macrophages, which are key players in inflammation, also influence bone regeneration. M1 macrophages produce inflammatory cytokines that negatively affect osteoblast activity, while M2 macrophages release anti-inflammatory factors that support osteoblast differentiation. Modulating immune responses is essential for balancing inflammation and bone formation. 66 Regulatory T cells become more active, modulating the immune response and preventing excessive inflammation. This transition is important for bone healing, enabling matrix breakdown, new tissue formation, and implant integration. A strong immune response can improve osseointegration, enhancing the success of implants and treatments. 67

HA plays an important role in inducing immune responses for osteogenesis. The material's physical properties, such as particle size, rough surface, porosity, and medium crystallinity (in ∼40–60%), will significantly influence the recognition of immune cells. 68 In the inflammatory phase, HA will easily induce the activation of macrophage through Toll-like receptor binding. This interaction triggers the release of proinflammatory cytokines, such as TNF-α, IL-1β, and IL-6. 37 Then, macrophages can undergo a polarization process into M1 and M2. M1 will be involved in the inflammatory phase to promote osteoclast differentiation and bone resorption, as well as eliminate pathogens and foreign substances after the defect. M2 will undergo the anti-inflammatory response to promote osteogenesis and reduce excessive inflammation at the site of implantation. 68 HA can also induce neutrophil migration and secrete the proinflammatory cytokine and enzyme to eliminate the pathogen. Meanwhile, neutrophils primarily contribute to inflammation, which plays a more substantial role in long-term osteogenesis. 69

## Conclusion

Osteogenesis is the dominant process in bone formation and is characterized by early bone development and terminalization in the extracellular matrix. Bone regeneration is a complex, gradual, and dynamic process. This process is initiated by the migration and induction of stem cells, which subsequently proliferate, differentiate, and induce matrix deposition. Factors that may determine the efficiency of osteogenesis are (1) the ratio of calcium and phosphorus concentrations, (2) porosity and pore size, (3) the ability of angiogenesis or induction to form new blood vessels, (4) the ability to modulate the immune system, and (5) the ability to induce growth hormone. Natural HA is one of many sources of HA. The use of BHA in several studies has shown good results, with some even better than synthetic HA. Therefore, HA can be a biomaterial for treating bone defects owing to its osteoconductive, osteoinductive, and osteointegrative properties, which trigger osteogenesis.
